# Decreases in GSH:GSSG activate vascular endothelial growth factor receptor 2 (VEGFR2) in human aortic endothelial cells

**DOI:** 10.1016/j.redox.2018.07.015

**Published:** 2018-07-25

**Authors:** Priya K. Prasai, Bandana Shrestha, A. Wayne Orr, Christopher B. Pattillo

**Affiliations:** a Department of Molecular and Cellular Physiology, Louisiana State University Health Sciences Center, Shreveport, LA 71130, USA; b Department of Cell Biology and Anatomy, Louisiana State University Health Sciences Center, Shreveport, LA 71130, USA; c Department of Pathology and Translational Pathobiology, Louisiana State University Health Sciences Center, Shreveport, LA 71130, USA

## Abstract

The angiogenic capacity of local tissue critically regulates the response to ischemic injury. Elevated reactive oxygen species production, commonly associated with ischemic injury, has been shown to promote phosphorylation of the vascular endothelial growth factor receptor 2 (VEGFR2), a critical regulator of angiogenesis. Previous data from our lab demonstrated that diminished levels of the antioxidant glutathione positively augment ischemic angiogenesis. Here, we sought to determine the relationship between glutathione levels and oxidative stress in VEGFR2 signaling. We reveal that decreasing the ratio of GSH to GSSG with diamide leads to enhanced protein S-glutathionylation, increased reactive oxygen species (ROS) production, and enhanced VEGFR2 activation. However, increasing ROS alone was insufficient in activating VEGFR2, while ROS enhanced VEGF-stimulated VEGFR2 activation at supraphysiological levels. We also found that inhibiting glutathione reductase activity is sufficient to increase VEGFR2 activation and sensitizes cells to ROS-dependent VEGFR2 activation. Taken together, these data suggest that regulation of the cellular GSH:GSSG ratio critically regulates VEGFR2 activation. This work represents an important first step in separating thiol mediated signaling events from ROS dependent signaling.

## Graphical abstract

Changes in GSH:GSSG but not ROS alone activates VEGFR2. This is a brief summary of the manuscript demonstrating that the addition of exogenous H_2_O_2_ is insufficient to activate VEGFR2; however decreasing GSH:GSSG results in ligand independent activation of VEGFR2.

**Figure:**
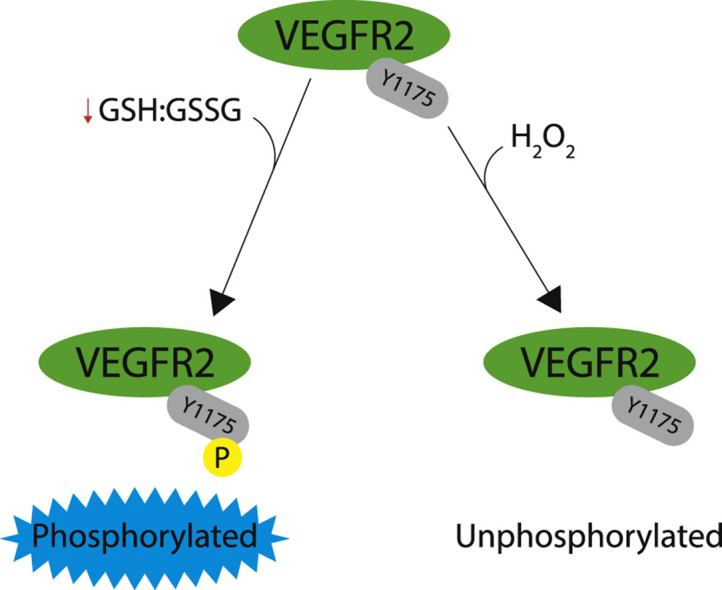

## 1 Introduction

Glutamate, cysteine, and glycine make up glutathione (GSH), the most abundant redox buffer in the cell. Scavenging metabolically derived reactive oxygen species (ROS) is a primary function of GSH. GSH is used in many enzymatic reactions to buffer ROS. One of the by-products of these enzymatically driven reactions involving GSH is oxidized glutathione (GSSG). GSSG is either recycled to GSH by the enzyme glutathione reductase (GR) or exported out of the cell to maintain the intracellular redox balance between GSH and GSSG, which, depending on cell type normally ranges from 200:1 – 30:1 [1]. In various pathologies the ratio is much lower with values as low as 1:1 reported in pediatric patients with anaplastic ependymomas [2].

The dynamic changes in the glutathione redox couple control several cellular processes including cell proliferation, differentiation, and apoptosis. Cellular processes are driven by thiol-disulfide switches or “nano-switches” (a phrase used by Schafer and Buettner), which are regulated by the GSH redox couple [3]. Oxidizing reactions shift the GSH:GSSG ratio lower as the cell proceeds from proliferation to differentiation and apoptosis [4]. Menon et al. demonstrated that transient increases in GSSG during the G1 phase of the cell cycle is a requirement for the cell to enter S phase [5]. Despite these data, there is still a paucity of information describing the effect of the GSH driven redox state on growth factor receptor signaling.

Glutathione has been shown to regulate protein signaling through a process called S-glutathionylation, a redox-dependent post-translational modification. A potential mechanism to stimulate S-glutathionylation is by increasing intracellular GSSG levels, allowing modification of cysteine residues through a thiol-disulfide exchange [6], [7]. Changes in the intracellular GSH redox state will affect the thiol-disulfide status of the cell and result in altered cellular signaling. The GSH:GSSG ratio changes in many cardiovascular diseases [8], such as peripheral vascular disease [9]. The ratio change can be attributed to tissue hypoxia decreasing GSH levels, which lead to a corresponding increase in GSSG levels.

Our lab previously demonstrated that altering basal glutathione levels affects angiogenesis in a murine model of ischemic vascular remodeling through an increase in VEGF production [10]. We showed that decreased GSH led to increased ROS and VEGF production and robust angiogenesis. Strangely further decreasing GSH led to more ROS production but not VEGF production and resulted in blunted angiogenesis. These data suggest that GSH:GSSG levels and not ROS regulate VEGFR2 signaling. The experiments in this manuscript are focused on elucidating the role of the GSH:GSSG ratio in VEGFR2 signaling.

## 2 Materials and methods

### 2.1 Cell culture

Human aortic endothelial cells (HAEC) were purchased from Lonza at p3 and sub-cultured in MCDB 131 media supplemented with bovine brain extract, fetal bovine serum, and heparin. HAECs were propagated at 37 °C in 5% CO2. All experiments were performed between passages 7–10.

### 2.2 Western blotting

Briefly, a confluent endothelial monolayer was serum depleted (0.5%) for 20 h, then treated with increasing concentrations (0, 50, 100, 150, 250 and 375 μM) of diamide or H_2_O_2_ (0, 10, 50, 100, 200, and 1000 μM) for 30 min. The experimental groups combining diamide or H_2_O_2_ with VEGF-A treatment received VEGF-A after 15 min of diamide or H_2_O_2_ treatment. Cell groups treated with peg-catalase were preloaded for 16 h with 500 μM before addition of diamide. Cells were then collected in 2X laemmli buffer. The cell lysate was boiled at 95 °C and stored at −80 °C until use. Cell lysates were separated using a 7.5% SDS gel and transferred to PVDF membranes. Antibodies used were phospho Y1175 VEGFR2, total VEGFR2, β-tubulin (Cell Signaling), total glutathionylation antibody (EMD Millipore) and secondary antibodies from Cell Signaling and Jackson ImmunoResearch.

### 2.3 High-pressure liquid chromatography (HPLC)

High-performance liquid chromatography (HPLC) was employed to measure GSH and GSSG using a previously described technique [11]. Briefly, for total GSH measurements, the cell culture monolayer with or without treatment was washed with ice-cold PBS, lysed and protein precipitated using 5% trichloroacetic acid (TCA) (GSH levels change if cells are pelleted first then acid precipitated; data not shown). Cell culture media was also collected and acid precipitated with an equal volume of 10% TCA (for a final volume of 5% TCA). The acid precipitate was centrifuged and the supernatant was used for independent GSH and GSSG measurements.

### 2.4 ROS assay

CM-H_2_DCF-DA (Molecular Probes) was used to measure total intracellular ROS production with and without diamide or H_2_O_2_ treatment using manufacturer's protocol. Briefly, cells were starved overnight, washed, loaded with dye for 30 min at 37 °C and then treated with either diamide, positive control (H_2_O_2_), or vehicle for another 30 min at 37 °C. Fluorescence intensity (492ex/527em) of each of the samples was normalized to control to calculate fold-change. CM-H2DCF-DA should not be used as a measure of specific species of ROS, but rather as a pan-ROS indicator. Kalyanaraman et al. [12] published a nice review indicating the caveats of using DCF as an ROS indicator.

### 2.5 ELISA

The concentration of VEGF-A (VEGF_165_) released into the media after diamide stimulation was quantified using a human VEGF quantikine ELISA kit (R&D systems) following the manufacturer's protocol.

### 2.6 Statistical Analysis

All experimental data were collected in triplicates and one-way ANOVA with Bonferroni correction was used to analyze data using GraphPad Prism software version 6.0. A p value less than 0.05 was considered significant.

## 3 Results

### 3.1 Diamide alters GSH redox state

There are three commonly used chemicals to alter GSH:GSSG; these compounds are buthionine sulfoximine (BSO), 1,3-bis-(2-chloroethyl)-1-nitrosourea (BCNU), and diamide. BSO selectively inhibits gamma-glutamyl cysteinyl ligase and inhibits de novo formation of GSH but has been shown to not directly affect GSH:GSSG [13]; this compound will likely lead to a decrease in GSH:GSSG over time as physiologic ROS production and GSH oxidation occur. BCNU is commonly used to inhibit GR, and as a consequence will decrease GSH:GSSG; however it is not as efficient as 2-AAPA and can also alkylate DNA [14]. For this study we used diamide, a cell-permeable thiol oxidant, reacts with GSH and chemically oxidizes it to glutathione disulfide (GSSG) in a predicted stoichiometric ratio; 0.5 mol of diamide is required to oxidize 1 mol of GSH [15]. HAECs were exposed to increasing concentrations of diamide (0, 50, 100, 150, 250 and 375 μM) for 30 min at 37 °C. Proteins were precipitated and intracellular concentrations as well as media concentrations were used to measure GSH and GSSG.

**Fig. 1:**
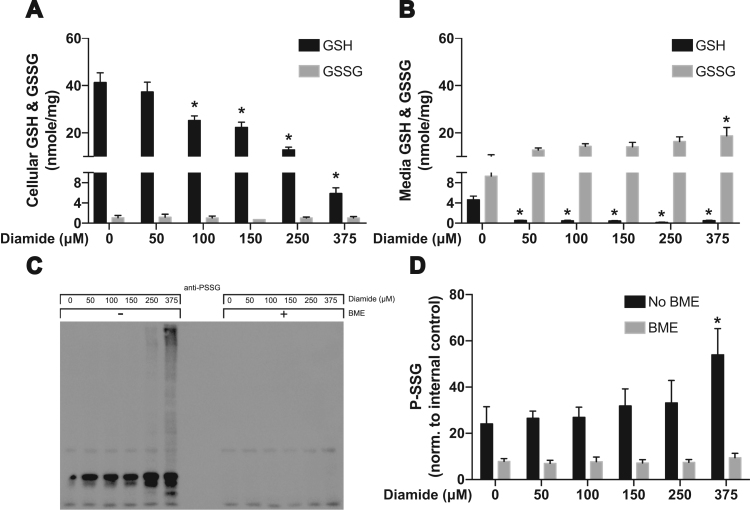
GSH and GSSG levels after redox alteration using diamide. **(A)** Intracellular concentrations and **(B)** extracellular concentrations of GSH and GSSG were measured using HPLC following 30 min of diamide treatment [* = p < 0.05 compared to respective control]. **(C)** Total S-glutathionylation of proteins after 30 min of diamide treatment [* = p < 0.05 compared to respective control].

Diamide exposure resulted in a dose-dependent depletion of intracellular GSH while the concentrations of GSSG did not show significant changes among the treatment groups (Fig. 1A). GSSG losses may occur through export into the extracellular space and/or binding to intracellular proteins through S-glutathionylation. To determine the likely fate of GSSG in our model, we first investigated the release of GSSG by measuring extracellular GSH and GSSG. We observed a trend for increased levels of GSSG in the culture medium following exposure to diamide (Fig. 1B) as well as a significant decrease in extracellular GSH. Following this finding we also measured total S-glutathionylation using Western Blot. We found a significant increase in total S-glutathionylated proteins exposed to 375 μM diamide compared to the 0, 50, 100 and 150 μM treated groups (Fig. 1C and D). In these experiments β-mercaptoethanol was used to reduce disulfide bonds and verify the specificity of the antibody for glutathionylated proteins.

### 3.2 Diamide activates VEGFR2

**Fig. 2:**
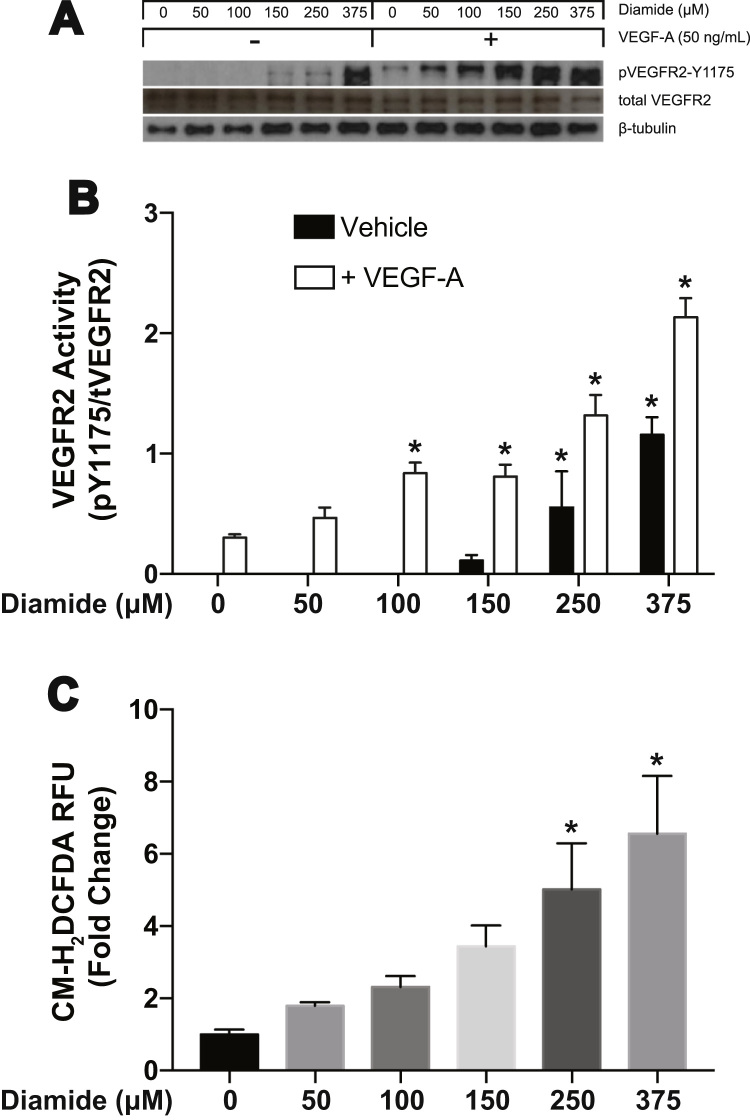
Diamide activates VEGFR2 and potentiates VEGF-A mediated receptor activation. **(A)** Western blot analysis for VEGFR2 phosphorylation at Y1175 after 15 min of diamide treatment followed by 15 min of VEGF-A treatment. [* = p < 0.0001 compared to control respective control]. **(B)** CM-H_2_DCFDA was used to measure the total cellular ROS production after subjecting HAECs to diamide treatment for 30 min [* = p < 0.0001 compared to control].

To study the molecular mechanism that led to enhanced vascularity *in vivo*, we used diamide as a tool to oxidize GSH in our *in vitro* system using human aortic endothelial cells. Endothelial cell proliferation and migration, important for blood vessel remodeling, is driven by complex signaling pathways initiated by VEGF binding to VEGFR2. To determine if changes in the GSH redox state in endothelial cells affects VEGF-dependent VEGFR2 activation, we assessed the phosphorylation state of VEGFR2 at tyrosine (Y) 1175 following treatment with increasing concentrations of diamide. Phosphorylation of VEGFR2 at Y1175 is important to trigger downstream signaling through recruitment of phospholipase Cγ (PLCγ) and phosphatidyl inositol 3-phosphate kinase (PI3K) leading to both proliferation and migration of endothelial cells. Our results show that at all concentrations of diamide VEGF-dependent VEGFR2 activation is enhanced (Fig. 2A and B). Interestingly, in the absence of exogenous VEGF, diamide also significantly activates VEGFR2 at concentrations of 250 and 375 μM (Fig. 2A and B).

To explore the possibility that VEGFR2 activation occurs through diamide by increasing reactive oxygen species (ROS) levels, we measured the release of ROS in HAECs following diamide treatment. We used CM-H_2_DCFDA to provide an idea of total overall ROS production. Our data shows a dose-dependent increase in total cellular ROS after diamide treatment (Fig. 2C).

### 3.3 Increased intracellular reactive oxygen species is not sufficient to activate VEGFR2

**Fig. 3:**
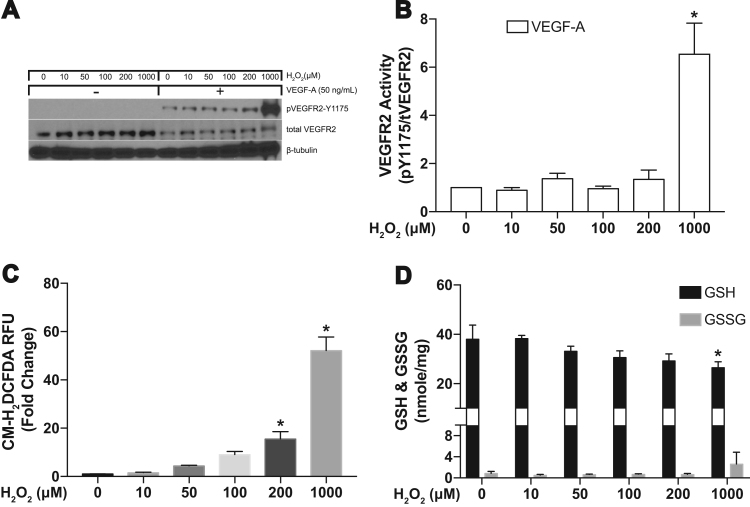
Hydrogen peroxide does not activate VEGFR2. **(A** and **B)** Western blot analysis for VEGFR2 phosphorylation at Y1175 after 15 min of H_2_O_2_ treatment followed 15 min of VEGF-A treatment. [* = p < 0.05 compared to control] **(C)** CM-H_2_DCFDA was used to measure the total cellular ROS production after subjecting HAECs to H_2_O_2_ for 30 min [* = p < 0.0001 compared to control]. **(D)** Intracellular concentrations of GSH and GSSG were measured using HPLC following 30 min of H_2_O_2_ treatment [* = p < 0.05 compared to respective control].

Following our finding that diamide increases ROS production we wanted to verify increased ROS activates VEGFR2. Therefore the next sets of experiments were designed to determine if exogenous addition of H_2_O_2_ alone is sufficient to activate VEGFR2. We treated HAECs with increasing concentrations of H_2_O_2_ (0, 10, 50, 100, 200, and 1000 μM) for 30 min. Similar to our experiments using diamide treatment, we treated HAECs with and without VEGF-A and tested for VEGFR2 activation using Y1175 as our readout. The results show that H_2_O_2_ alone does not activate the receptor and does not enhance VEGF-dependent VEGFR2 activation until concentrations reach 1 mM (Fig. 3A and B). To confirm exogenous H_2_O_2_ treatment increases ROS concentrations we used CM-H_2_DCFDA. Fig. 3C shows increasing H_2_O_2_ correlates with increasing ROS levels. To test if the concentrations of H_2_O_2_ used are sufficient to change GSH redox state, we also measured intracellular concentrations of GSH and GSSG. We observed a significant decrease in intracellular concentrations of GSH only at the 1 mM concentration of H_2_O_2_, a value that is far outside of a physiologic level (Fig. 3D).

### 3.4 Increase in cellular GSSG is required for VEGFR2 activation under altered GSH redox state

**Fig. 4:**
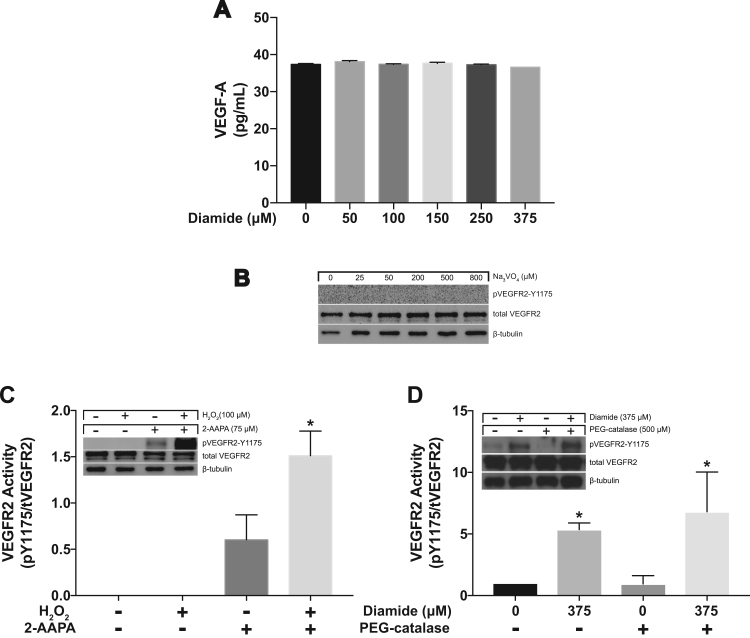
Diamide does not stimulate VEGF release and inhibiting PTPs is insufficient to stimulate VEGFR2 activation. **(A)** VEGF-A was measured using ELISA following 30 min of diamide treatment. **(B)** Western blot analysis following 30 min of sodium orthovanadate (Na_3_VO_4_) treatment (to inhibit PTP activity). **(C)** VEGFR2 phosphorylation at Y1175 following 30 min of 2-AAPA treatment (to inhibit GR activity) followed by 30 min of H_2_O_2_ treatment. [* = p < 0.05 compared to 2-AAPA] **(D)** VEGFR2 phosphorylation at Y1175 following 16 h pretreatment with peg-catalase followed by diamide treatment for 30 min. [* = p < 0.05 0 μM diamide compared to 375 μM diamide].

Additionally, we wanted to verify that our use of diamide was not inadvertently increasing VEGF production or release. Cell media was collected 30 min following diamide treatment and VEGF was measured using an ELISA kit. There was no change in the amount of VEGF in the media despite increasing levels of diamide (Fig. 4A). Protein tyrosine phosphatase (PTP) activity can be inactivated by GSSG [16] so we next inhibited PTPs to determine if inhibition alone would result in activation of VEGFR2. We used increasing concentrations of sodium orthovanadate (Na_3_VO_4_) to inhibit PTPs and found no detectable activation of VEGFR2 (Fig. 4B).

It is well established that diamide enhances protein S-glutathionylation [17] and we have demonstrated the same in HAECs (Fig. 1C and D). To confirm the involvement of GSSG in diamide-induced VEGFR2 activation, we used 2-AAPA, a dithiocarbamate derivative, to inhibit glutathione reductase activity (GR) [14]. In our HAEC cells the GR activity (measured with the GR assay kit; Cayman Chemicals, MI) was 7.05 ± 0.64 (SD) in control cells and undetectable in 2-AAPA treated cells (75 μM). As mentioned earlier, GR is an enzyme in the GSH cycle that converts oxidized glutathione (GSSG) to reduced glutathione (GSH). Inhibition of GR will result in the accumulation of GSSG creating favorable conditions for protein S-glutathionylation. We pretreated the cells with 75 μM of 2-AAPA for 30 min and then added 100 μM of H_2_O_2_. VEGFR2 was phosphorylated at Y1175 in the 2-AAPA only and 2-AAPA + H_2_O_2_ combination treatment (Fig. 4C). It is important to note that H_2_O_2_ alone does not activate the receptor. This shows the potential role for GSSG in the activation of VEGFR2.

Finally we verified that H_2_O_2_ was not being produced downstream of our diamide treatment by pre-treating HAECs with peg-catalase (Fig. 4D). We already demonstrated that exogenous addition of H_2_O_2_ was insufficient to activate VEGFR2 in our model cells (HAECs) in Fig. 3. Together, these results show that VEGFR2 is regulated by the GSH redox state of the cell and is not dependent solely on ROS production.

## 4 Discussion

VEGFR2 signaling is essential to vascular development, under both pathologic and physiologic conditions. The production of ROS also plays a major role in vascular development, with the addition of antioxidants such as N-Acetyl Cysteine unsurprisingly interrupting the majority of signaling pathways, due to their ability to scavenge ROS. This approach to treat disease is a simplistic view of a major signaling dynamic. We believe a major determinant in angiogenic signaling pathways is the local cellular redox environment. Endogenous antioxidants, such as GSH, are less well studied in the context of signaling during vessel growth, however research in this area is rapidly expanding.

Two studies have demonstrated ligand independent VEGFR2 activation illustrating potential alternate signaling pathways. Jin et al. showed that shear stress in bovine arterial cells activated VEGFR2 in a ligand independent manner [18]. This activation could be induced through NADPH oxidase production of ROS in response to shear [19] and is a logical lead in to the more recent study by Warren et al. demonstrating that hydrogen peroxide activates VEGFR2 independent of ligand in human umbilical vein endothelial cells [20]. In our current study we have demonstrated that hydrogen peroxide alone (exogenous addition of concentrations as high as 1 mM) is insufficient to activate VEGFR2 in human arterial endothelial cells; however the addition of VEGF in the presence of exogenous hydrogen peroxide results in a robust increase in VEGFR2 activation over VEGF alone. Throughout our treatments VEGF levels were constant, as measured by ELISA. These studies shed light on a possible explanation for anti-VEGF therapies not being as effective in some instances clinically for the treatment of pathological angiogenesis as first hoped [21]. During states of increased oxidative stress the VEGFR2 receptor may still be activated despite having the ligand scavenged from the system.

This is the first study to demonstrate the regulation of VEGFR2 by the glutathione redox state (Figure 5). Glutathione exists in the cell in both reduced (GSH) and oxidized (GSSG) forms that constitute the major redox couple of the cell [3]. Alteration changing this major redox couple can result in significant changes to the internal environment of the cell and resultant GSSG increases can trigger S-glutathionylation [17], [22] of key proteins. In this study we have clearly demonstrated that decreases in GSH:GSSG and not ROS alone allow the activation of VEGFR2 in human aortic endothelial cells.

These data support the hypothesis that arterial signaling is regulated in a thiol dependent manner. This study opens an avenue to more thoroughly understand VEGFR2 signaling that was previously thought to rely heavily on alterations in ROS production and the subsequent cysteine oxidation steps leading to various protein activity changes. Quite possibly the more important signaling contributors are the available thiols, including but not limited to glutathione, hydrogen sulfide, and persulfide pools in the cell. These thiol pools fluctuate during many disease states and may or may not need ROS changes to contribute to protein modifications.

A clear limitation of the current study is a lack of specific mechanisms driving the activation of VEGFR2. Our lab is currently exploring several avenues of activation. VEGFR2 appears to be glutathionylated however the modification is transient and finding the specific timepoint of glutathionylation has been challenging. Another potential mechanism of activation is in the Src pathway. Studies of both mechanisms are currently underway.
